# Supplementary material for: Nasal Bacteriomes of Patients with Asthma and Allergic Rhinitis Show Unique Composition, Structure, Function and Interactions
Source: Microorganisms. 2023 Mar 7;11(3):683. doi: 10.3390/microorganisms11030683 (PMC10056468; doi:10.3390/microorganisms11030683)
Supplement: Supplementary file 1 [file microorganisms-11-00683-s001.zip › Figure S3.pdf]

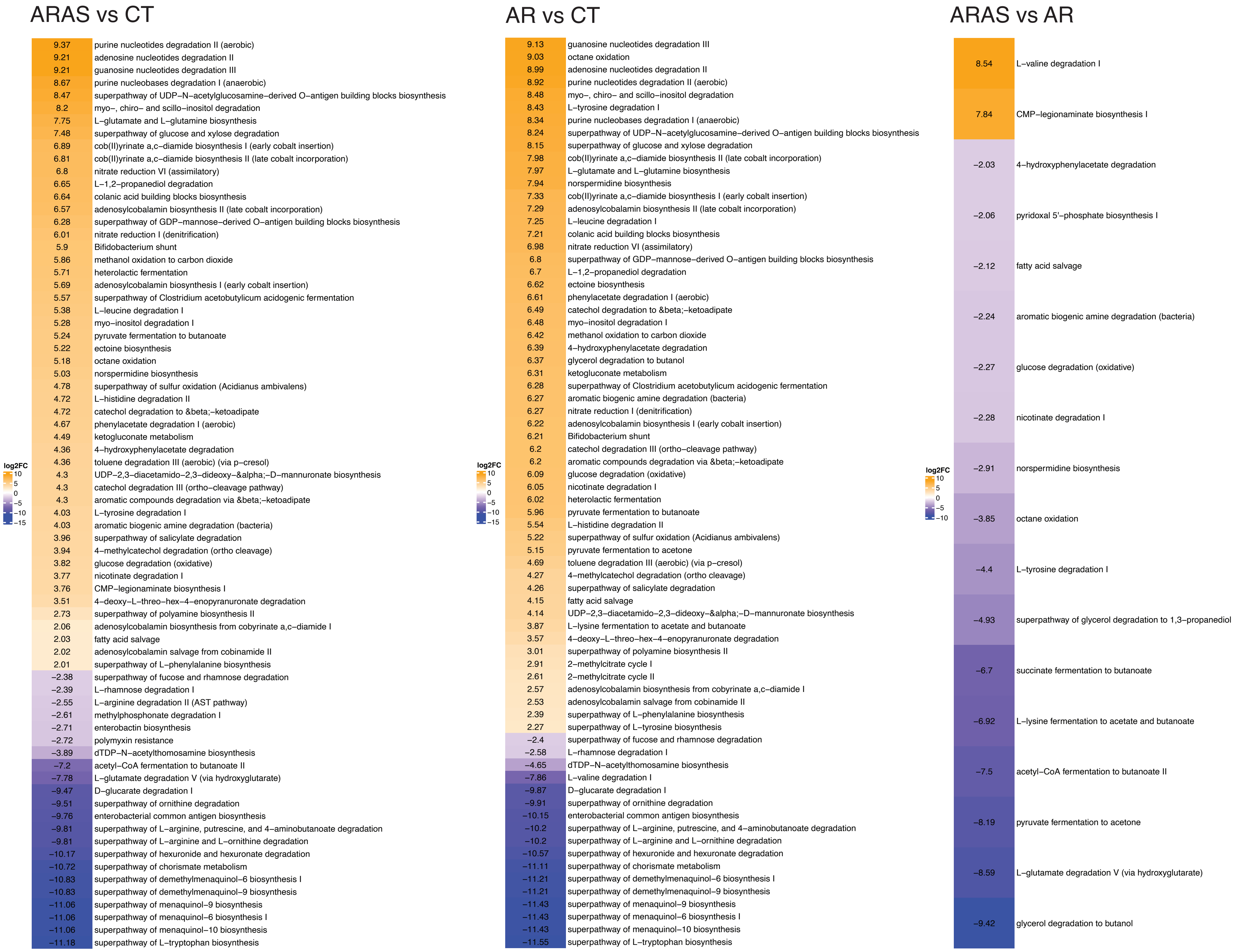

ARAS vs CT
9.37 purine nucleotides degradation II (aerobic)
9.21 adenosine nucleotides degradation II
9.21 guanosine nucleotides degradation III
8.67 purine nucleobases degradation I (anaerobic)
8.47 superpathway of UDP−N−acetylglucosamine−derived O−antigen building blocks biosynthesis
8.2 myo−, chiro− and scillo−inositol degradation
7.75 L−glutamate and L−glutamine biosynthesis
7.48 superpathway of glucose and xylose degradation
6.89 cob(II)yrinate a,c−diamide biosynthesis I (early cobalt insertion)
6.81 cob(II)yrinate a,c−diamide biosynthesis II (late cobalt incorporation)
6.8 nitrate reduction VI (assimilatory)
6.65 L−1,2−propanediol degradation
6.64 colanic acid building blocks biosynthesis
6.57 adenosylcobalamin biosynthesis II (late cobalt incorporation)
6.28 superpathway of GDP−mannose−derived O−antigen building blocks biosynthesis
6.01 nitrate reduction I (denitrification)
5.9 Bifidobacterium shunt
5.86 methanol oxidation to carbon dioxide
5.71 heterolactic fermentation
5.69 adenosylcobalamin biosynthesis I (early cobalt insertion)
5.57 superpathway of Clostridium acetobutylicum acidogenic fermentation
5.38 L−leucine degradation I
5.28 myo−inositol degradation I
5.24 pyruvate fermentation to butanoate
5.22 ectoine biosynthesis
5.18 octane oxidation
5.03 norspermidine biosynthesis
4.78 superpathway of sulfur oxidation (Acidianus ambivalens)
4.72 L−histidine degradation II
4.72 catechol degradation to β−ketoadipate
4.67 phenylacetate degradation I (aerobic)
4.49 ketogluconate metabolism
4.36 4−hydroxyphenylacetate degradation
4.36 toluene degradation III (aerobic) (via p−cresol)
4.3 UDP−2,3−diacetamido−2,3−dideoxy−α−D−mannuronate biosynthesis
4.3 catechol degradation III (ortho−cleavage pathway)
4.3 aromatic compounds degradation via β−ketoadipate
4.03 L−tyrosine degradation I
4.03 aromatic biogenic amine degradation (bacteria)
3.96 superpathway of salicylate degradation
3.94 4−methylcatechol degradation (ortho cleavage)
3.82 glucose degradation (oxidative)
3.77 nicotinate degradation I
3.76 CMP−legionaminate biosynthesis I
3.51 4−deoxy−L−threo−hex−4−enopyranuronate degradation
2.73 superpathway of polyamine biosynthesis II
2.06 adenosylcobalamin biosynthesis from cobyrinate a,c−diamide I
2.03 fatty acid salvage
2.02 adenosylcobalamin salvage from cobinamide II
2.01 superpathway of L−phenylalanine biosynthesis
−2.38 superpathway of fucose and rhamnose degradation
−2.39 L−rhamnose degradation I
−2.55 L−arginine degradation II (AST pathway)
−2.61 methylphosphonate degradation I
−2.71 enterobactin biosynthesis
−2.72 polymyxin resistance
−3.89 dTDP−N−acetylthomosamine biosynthesis
−7.2 acetyl−CoA fermentation to butanoate II
−7.78 L−glutamate degradation V (via hydroxyglutarate)
−9.47 D−glucarate degradation I
−9.51 superpathway of ornithine degradation
−9.76 enterobacterial common antigen biosynthesis
−9.81 superpathway of L−arginine, putrescine, and 4−aminobutanoate degradation
−9.81 superpathway of L−arginine and L−ornithine degradation
−10.17 superpathway of hexuronide and hexuronate degradation
−10.72 superpathway of chorismate metabolism
−10.83 superpathway of demethylmenaquinol−6 biosynthesis I
−10.83 superpathway of demethylmenaquinol−9 biosynthesis
−11.06 superpathway of menaquinol−9 biosynthesis
−11.06 superpathway of menaquinol−6 biosynthesis I
−11.06 superpathway of menaquinol−10 biosynthesis
−11.18 superpathway of L−tryptophan biosynthesis
log2FC
10
5
0
−5
−10
−15
AR vs CT
9.13 guanosine nucleotides degradation III
9.03 octane oxidation
8.99 adenosine nucleotides degradation II
8.92 purine nucleotides degradation II (aerobic)
8.48 myo−, chiro− and scillo−inositol degradation
8.43 L−tyrosine degradation I
8.34 purine nucleobases degradation I (anaerobic)
8.24 superpathway of UDP−N−acetylglucosamine−derived O−antigen building blocks biosynthesis
8.15 superpathway of glucose and xylose degradation
7.98 cob(II)yrinate a,c−diamide biosynthesis II (late cobalt incorporation)
7.97 L−glutamate and L−glutamine biosynthesis
7.94 norspermidine biosynthesis
7.33 cob(II)yrinate a,c−diamide biosynthesis I (early cobalt insertion)
7.29 adenosylcobalamin biosynthesis II (late cobalt incorporation)
7.25 L−leucine degradation I
7.21 colanic acid building blocks biosynthesis
6.98 nitrate reduction VI (assimilatory)
6.8 superpathway of GDP−mannose−derived O−antigen building blocks biosynthesis
6.7 L−1,2−propanediol degradation
6.62 ectoine biosynthesis
6.61 phenylacetate degradation I (aerobic)
6.49 catechol degradation to β−ketoadipate
6.48 myo−inositol degradation I
6.42 methanol oxidation to carbon dioxide
6.39 4−hydroxyphenylacetate degradation
6.37 glycerol degradation to butanol
6.31 ketogluconate metabolism
6.28 superpathway of Clostridium acetobutylicum acidogenic fermentation
6.27 aromatic biogenic amine degradation (bacteria)
6.27 nitrate reduction I (denitrification)
6.22 adenosylcobalamin biosynthesis I (early cobalt insertion)
6.21 Bifidobacterium shunt
6.2 catechol degradation III (ortho−cleavage pathway)
6.2 aromatic compounds degradation via β−ketoadipate
6.09 glucose degradation (oxidative)
6.05 nicotinate degradation I
6.02 heterolactic fermentation
5.96 pyruvate fermentation to butanoate
5.54 L−histidine degradation II
5.22 superpathway of sulfur oxidation (Acidianus ambivalens)
5.15 pyruvate fermentation to acetone
4.69 toluene degradation III (aerobic) (via p−cresol)
4.27 4−methylcatechol degradation (ortho cleavage)
4.26 superpathway of salicylate degradation
4.15 fatty acid salvage
4.14 UDP−2,3−diacetamido−2,3−dideoxy−α−D−mannuronate biosynthesis
3.87 L−lysine fermentation to acetate and butanoate
3.57 4−deoxy−L−threo−hex−4−enopyranuronate degradation
3.01 superpathway of polyamine biosynthesis II
2.91 2−methylcitrate cycle I
2.61 2−methylcitrate cycle II
2.57 adenosylcobalamin biosynthesis from cobyrinate a,c−diamide I
2.53 adenosylcobalamin salvage from cobinamide II
2.39 superpathway of L−phenylalanine biosynthesis
2.27 superpathway of L−tyrosine biosynthesis
−2.4 superpathway of fucose and rhamnose degradation
−2.58 L−rhamnose degradation I
−4.65 dTDP−N−acetylthomosamine biosynthesis
−7.86 L−valine degradation I
−9.87 D−glucarate degradation I
−9.91 superpathway of ornithine degradation
−10.15 enterobacterial common antigen biosynthesis
−10.2 superpathway of L−arginine, putrescine, and 4−aminobutanoate degradation
−10.2 superpathway of L−arginine and L−ornithine degradation
−10.57 superpathway of hexuronide and hexuronate degradation
−11.11 superpathway of chorismate metabolism
−11.21 superpathway of demethylmenaquinol−6 biosynthesis I
−11.21 superpathway of demethylmenaquinol−9 biosynthesis
−11.43 superpathway of menaquinol−9 biosynthesis
−11.43 superpathway of menaquinol−6 biosynthesis I
−11.43 superpathway of menaquinol−10 biosynthesis
−11.55 superpathway of L−tryptophan biosynthesis
log2FC
10
5
0
−5
−10
−15
ARAS vs AR
8.54 L−valine degradation I
7.84 CMP−legionaminate biosynthesis I
−2.03 4−hydroxyphenylacetate degradation
−2.06 pyridoxal 5'−phosphate biosynthesis I
−2.12 fatty acid salvage
−2.24 aromatic biogenic amine degradation (bacteria)
−2.27 glucose degradation (oxidative)
−2.28 nicotinate degradation I
−2.91 norspermidine biosynthesis
−3.85 octane oxidation
−4.4 L−tyrosine degradation I
−4.93 superpathway of glycerol degradation to 1,3−propanediol
−6.7 succinate fermentation to butanoate
−6.92 L−lysine fermentation to acetate and butanoate
−7.5 acetyl−CoA fermentation to butanoate II
−8.19 pyruvate fermentation to acetone
−8.59 L−glutamate degradation V (via hydroxyglutarate)
−9.42 glycerol degradation to butanol
log2FC
10
5
0
−5
−10
