# Supplementary figures and images for: Nasal Bacteriomes of Patients with Asthma and Allergic Rhinitis Show Unique Composition, Structure, Function and Interactions
Source: Microorganisms. 2023 Mar 7;11(3):683. doi: 10.3390/microorganisms11030683 (PMC10056468; doi:10.3390/microorganisms11030683)

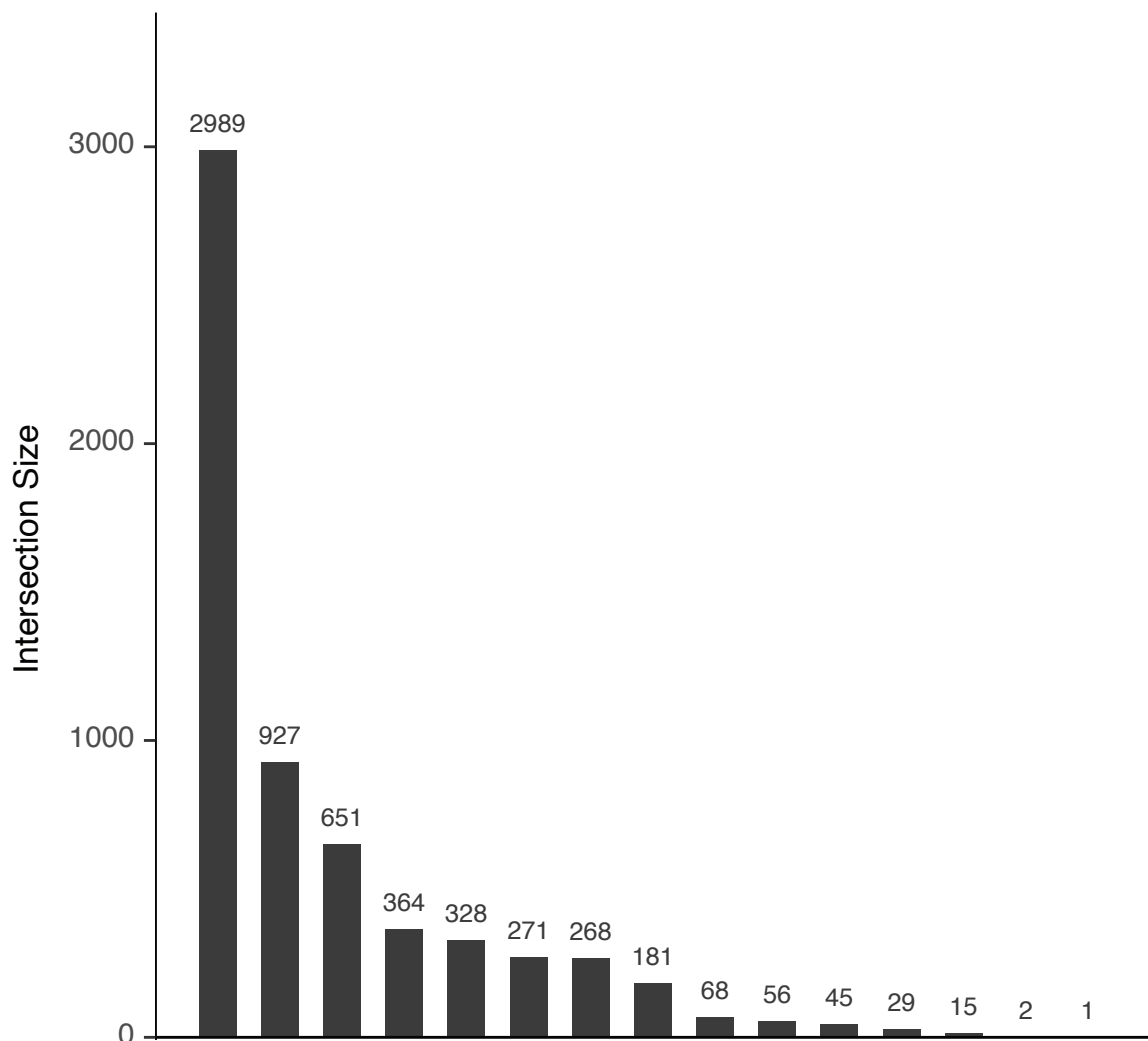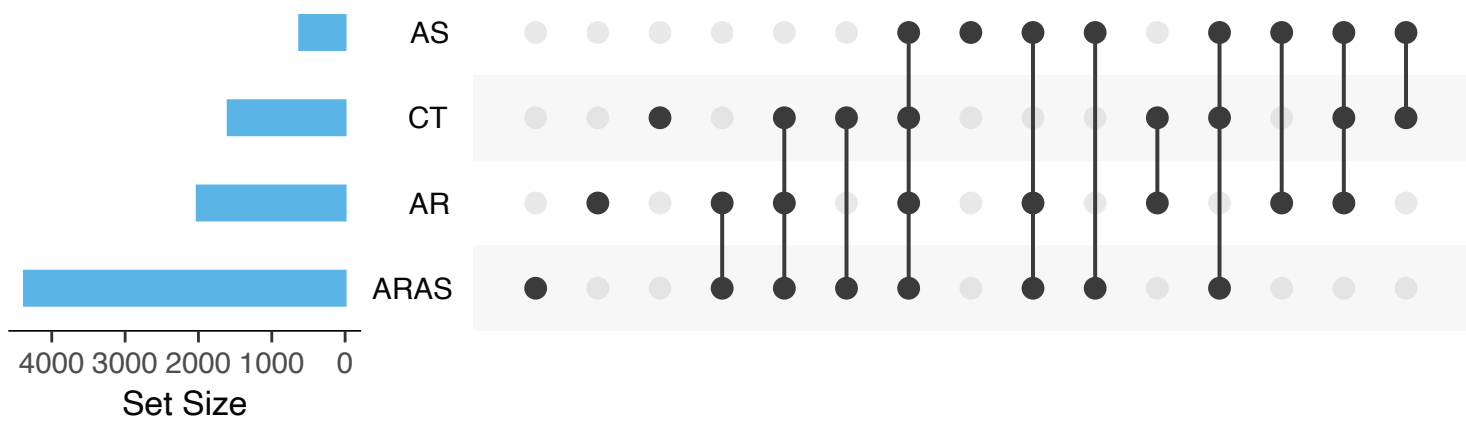

Supplement: Supplementary file 1 [file microorganisms-11-00683-s001.zip › Figure S2.pdf]
