# Supplementary material for: Nasal Bacteriomes of Patients with Asthma and Allergic Rhinitis Show Unique Composition, Structure, Function and Interactions
Source: Microorganisms. 2023 Mar 7;11(3):683. doi: 10.3390/microorganisms11030683 (PMC10056468; doi:10.3390/microorganisms11030683)

Nasal bacteriomes

2022-12-27

- Packages loading
- Filter samples and ASVs
- Core Microbiome
- Data normalization
- Estimate alpha-diversity including pd
- Estimate beta-diversity
- PCoA plots
- PICRUSt2 commands
  - From these last objects, prepare log2foldchange vectors
- Arrange phyloseq objects for network analysis
- Networks calculation using neighborhood selection (mb) method
- Analyze the networks
  - Label the networks nodes by the best hit and remove unwanted characters
- Plot nasal bacteriome networks and color nodes by clusters/modules

Packages loading

```
library(phyloseq)
library(cowplot)
library(picante)
library(TSA)
library(multcomp)
library(microbiome)
library(mvabund)
library(geepack)
library(doBy)
library(lattice)
library(MuMIn)
library("DESeq2")
library(tidyverse)
library(stringr)
library(fantaxtic)
```

```
readRDS(file = "nasal_bacteriome.RDS") -> ps1
```

```
ps1
```

```
## phyloseq-class experiment-level object
## otu_table()   OTU Table:         [ 7406 taxa and 347 samples ]
## sample_data() Sample Data:       [ 347 samples by 6 sample variables ]
## tax_table()   Taxonomy Table:    [ 7406 taxa by 7 taxonomic ranks ]
## phy_tree()    Phylogenetic Tree: [ 7406 tips and 7405 internal nodes ]
```

Filter samples and ASVs

remove singletons

```
ps1 <- prune_taxa(taxa_sums(ps1) > 1, ps1)
ps1
```

```
## phyloseq-class experiment-level object
## otu_table()   OTU Table:         [ 6195 taxa and 347 samples ]
## sample_data() Sample Data:       [ 347 samples by 6 sample variables ]
## tax_table()   Taxonomy Table:    [ 6195 taxa by 7 taxonomic ranks ]
## phy_tree()    Phylogenetic Tree: [ 6195 tips and 6194 internal nodes ]
```

remove samples with less than 1000 reads

```
ps1 = prune_samples(sample_sums(ps1) >= 1000, ps1)
ps1
```

```
## phyloseq-class experiment-level object
## otu_table()   OTU Table:         [ 6195 taxa and 344 samples ]
## sample_data() Sample Data:       [ 344 samples by 6 sample variables ]
## tax_table()   Taxonomy Table:    [ 6195 taxa by 7 taxonomic ranks ]
## phy_tree()    Phylogenetic Tree: [ 6195 tips and 6194 internal nodes ]
```

```
min(colSums(otu_table(ps1)))
```

```
## [1] 1771
```

```
summarize_phyloseq(ps1)
```

```
## Compositional = NO2
```

```
## 1] Min. number of reads = 17712] Max. number of reads = 824303] Total number of reads = 65156094] Average numb
er of reads = 18940.72383720935] Median number of reads = 18245.57] Sparsity = 0.9860793588227576] Any OTU sum to
1 or less? NO8] Number of singletons = 09] Percent of OTUs that are singletons
##         (i.e. exactly one read detected across all samples)010] Number of sample variables are: 6patientseason
yearagesexasthma_rhinitis12
```

```
## [[1]]
## [1] "1] Min. number of reads = 1771"
##
## [[2]]
## [1] "2] Max. number of reads = 82430"
##
## [[3]]
## [1] "3] Total number of reads = 6515609"
##
## [[4]]
## [1] "4] Average number of reads = 18940.7238372093"
##
## [[5]]
## [1] "5] Median number of reads = 18245.5"
##
## [[6]]
## [1] "7] Sparsity = 0.986079358822757"
##
## [[7]]
## [1] "6] Any OTU sum to 1 or less? NO"
##
## [[8]]
## [1] "8] Number of singletons = 0"
##
## [[9]]
## [1] "9] Percent of OTUs that are singletons \n        (i.e. exactly one read detected across all samples)0"
##
## [[10]]
## [1] "10] Number of sample variables are: 6"
##
## [[11]]
## [1] "patient"          "season"           "year"             "age"
## [5] "sex"              "asthma_rhinitis1"
```

Core Microbiome

```
ps1.core <- core(ps1, detection = 0, prevalence = 0.9)
core.taxa <- taxa(ps1.core);core.taxa
```

```
## [1] "ASV1" "ASV2"
```

```
class(core.taxa)
```

```
## [1] "character"
```

Get the taxonomy data

```
tax.mat <- tax_table(ps1.core)
tax.df <- as.data.frame(tax.mat)
```

Add the ASVs to last

```
tax.df$ASV <- rownames(tax.df)
```

Select taxonomy of only. Those ASVs that are core members based on the thresholds that were used.

```
core.taxa.class <- dplyr::filter(tax.df, rownames(tax.df) %in% core.taxa)
knitr::kable(head(core.taxa.class))
```

	Kingdom	Phylum	Class	Order	Family	Genus	Species	ASV
ASV1	Bacteria	Firmicutes	Bacilli	Lactobacillales	Streptococcaceae	Streptococcus	oralis	ASV1
ASV2	Bacteria	Firmicutes	Bacilli	Staphylococcales	Staphylococcaceae	Staphylococcus	aureus	ASV2

```
knitr::kable(core.taxa.class)
```

	Kingdom	Phylum	Class	Order	Family	Genus	Species	ASV
ASV1	Bacteria	Firmicutes	Bacilli	Lactobacillales	Streptococcaceae	Streptococcus	oralis	ASV1
ASV2	Bacteria	Firmicutes	Bacilli	Staphylococcales	Staphylococcaceae	Staphylococcus	aureus	ASV2

estimate read proportions for the core

```
rank_names(ps1.core, errorIfNULL=TRUE)
```

```
## [1] "Kingdom" "Phylum"  "Class"   "Order"   "Family"  "Genus"   "Species"
```

```
taxon <- tax_glom(ps1.core, taxrank = "Species")
table_all<-cbind(tax_table(taxon),otu_table(taxon))
table_all_t<-t(data.matrix(table_all))
write.csv(table_all_t,file="core_N.csv")
summarize_phyloseq(ps1)
```

```
## Compositional = NO2
```

```
## 1] Min. number of reads = 17712] Max. number of reads = 824303] Total number of reads = 65156094] Average numb
er of reads = 18940.72383720935] Median number of reads = 18245.57] Sparsity = 0.9860793588227576] Any OTU sum to
1 or less? NO8] Number of singletons = 09] Percent of OTUs that are singletons
##         (i.e. exactly one read detected across all samples)010] Number of sample variables are: 6patientseason
yearagesexasthma_rhinitis12
```

```
## [[1]]
## [1] "1] Min. number of reads = 1771"
##
## [[2]]
## [1] "2] Max. number of reads = 82430"
##
## [[3]]
## [1] "3] Total number of reads = 6515609"
##
## [[4]]
## [1] "4] Average number of reads = 18940.7238372093"
##
## [[5]]
## [1] "5] Median number of reads = 18245.5"
##
## [[6]]
## [1] "7] Sparsity = 0.986079358822757"
##
## [[7]]
## [1] "6] Any OTU sum to 1 or less? NO"
##
## [[8]]
## [1] "8] Number of singletons = 0"
##
## [[9]]
## [1] "9] Percent of OTUs that are singletons \n        (i.e. exactly one read detected across all samples)0"
##
## [[10]]
## [1] "10] Number of sample variables are: 6"
##
## [[11]]
## [1] "patient"          "season"           "year"             "age"
## [5] "sex"              "asthma_rhinitis1"
```

Data normalization

```
diagdds = phyloseq_to_deseq2(ps1, ~season) # Any variable of the metadata would work to create the DESeq object
```

```
## converting counts to integer mode
```

```
## Warning in DESeqDataSet(se, design = design, ignoreRank): some variables in design formula
## are characters, converting to factors
```

Calculate geometric means

```
gm_mean = function(x, na.rm=TRUE){
  exp(sum(log(x[x > 0]), na.rm=na.rm) / length(x))
}
geoMeans = apply(counts(diagdds), 1, gm_mean)
```

Estimate size factors

```
diagdds = estimateSizeFactors(diagdds, geoMeans = geoMeans)
```

Get normalized read counts

```
normcounts <- counts(diagdds, normalized = TRUE)
```

Round read counts

```
round(normcounts, digits = 0) -> normcountsrd
```

Transform matrix of normalized counts to phyloseq object

```
otu_table(normcountsrd, taxa_are_rows = TRUE) -> ncr
```

Replace otu_table in original phyloseq object

```
otu_table(ps1) <- ncr
```

Estimate alpha-diversity including pd

```
otuD<-as.data.frame(t(otu_table(ps1)))
phylodiversityRAREF_Q<-pd(otuD, phy_tree(ps1), include.root=TRUE) ### Phylogenetic diversity. Include root=True t
ree rooted via midpoint
diversityRAREF_Q<-estimate_richness(ps1)
diversityRAREF_Q1<-cbind(sample_data(ps1),diversityRAREF_Q,phylodiversityRAREF_Q)
```

```
library(ggpubr)
```

```
my_comparisons <- list(c("AS", "CT"),c("AR", "CT"),c("ARAS", "CT"),c("AS", "AR"),c("AS", "ARAS"),c("AR", "ARAS"))
# List here the group pairs to compare statistically
```

```
compare_means(formula = Chao1~asthma_rhinitis1,data = diversityRAREF_Q1, method = "wilcox.test", exact= FALSE)
```

```
## # A tibble: 6 × 8
##   .y.   group1 group2            p     p.adj p.format p.signif method
##   <chr> <chr>  <chr>         <dbl>     <dbl> <chr>    <chr>    <chr>
## 1 Chao1 ARAS   AR     0.491        1         0.49     ns       Wilcoxon
## 2 Chao1 ARAS   AS     0.338        1         0.34     ns       Wilcoxon
## 3 Chao1 ARAS   CT     0.000000730 0.0000044 7.3e-07  ****     Wilcoxon
## 4 Chao1 AR     AS     0.344        1         0.34     ns       Wilcoxon
## 5 Chao1 AR     CT     0.0000591    0.0003    5.9e-05  ****     Wilcoxon
## 6 Chao1 AS     CT     0.526        1         0.53     ns       Wilcoxon
```

```
chao <- ggplot(diversityRAREF_Q1, aes(factor(asthma_rhinitis1), Chao1))
chao2<-chao + geom_boxplot(aes(fill = factor(asthma_rhinitis1)),outlier.colour = "black", outlier.size = 0.2)+ ge
om_jitter(size=0.2,shape=1)+panel_border(colour = "black", size = 0.5)+ ggtitle("Chao1 richness")+labs(y = "Chao1
richness") + stat_compare_means(mapping = NULL, comparisons = my_comparisons, hide.ns = FALSE, label = "p.signif"
,  label.x = NULL, label.y = NULL, exact =FALSE)
```

```
## [1] FALSE
```

```
compare_means(formula = Shannon~asthma_rhinitis1,data = diversityRAREF_Q1, method = "wilcox.test", exact= FALSE)
```

```
## # A tibble: 6 × 8
##   .y.     group1 group2        p  p.adj p.format p.signif method
##   <chr>   <chr>  <chr>     <dbl>  <dbl> <chr>    <chr>    <chr>
## 1 Shannon ARAS   AR     0.818    1      0.8175   ns       Wilcoxon
## 2 Shannon ARAS   AS     0.290    0.87   0.2896   ns       Wilcoxon
## 3 Shannon ARAS   CT     0.000100 0.0006 0.0001   ***      Wilcoxon
## 4 Shannon AR     AS     0.208    0.83   0.2078   ns       Wilcoxon
## 5 Shannon AR     CT     0.00263  0.013  0.0026   **       Wilcoxon
## 6 Shannon AS     CT     0.584    1      0.5844   ns       Wilcoxon
```

```
shan <- ggplot(diversityRAREF_Q1, aes(factor(asthma_rhinitis1), Shannon))
shan2<-shan + geom_boxplot(aes(fill = factor(asthma_rhinitis1)),outlier.colour = "black", outlier.size = 0.2)+ ge
om_jitter(size=0.2,shape=1)+panel_border(colour = "black", size = 0.5)+ ggtitle("Shannon diversity")+labs(y = "Sh
annon diversity") + stat_compare_means(mapping = NULL, comparisons = my_comparisons, hide.ns = FALSE, label = "p.
signif",  label.x = NULL, label.y = NULL, exact =FALSE)
```

```
## [1] FALSE
```

```
compare_means(formula = ACE~asthma_rhinitis1,data = diversityRAREF_Q1, method = "wilcox.test", exact= FALSE)
```

```
## # A tibble: 6 × 8
##   .y.   group1 group2            p     p.adj p.format p.signif method
##   <chr> <chr>  <chr>         <dbl>     <dbl> <chr>    <chr>    <chr>
## 1 ACE   ARAS   AR     0.382        1         0.38     ns       Wilcoxon
## 2 ACE   ARAS   AS     0.492        1         0.49     ns       Wilcoxon
## 3 ACE   ARAS   CT     0.000000461 0.0000028 4.6e-07  ****     Wilcoxon
## 4 ACE   AR     AS     0.428        1         0.43     ns       Wilcoxon
## 5 ACE   AR     CT     0.0000166    0.000083  1.7e-05  ****     Wilcoxon
## 6 ACE   AS     CT     0.320        1         0.32     ns       Wilcoxon
```

```
ACE <- ggplot(diversityRAREF_Q1, aes(factor(asthma_rhinitis1), ACE))
ACE2<- ACE + geom_boxplot(aes(fill = factor(asthma_rhinitis1)),outlier.colour = "black", outlier.size = 0.2)+ geo
m_jitter(size=0.2,shape=1)+panel_border(colour = "black", size = 0.5)+ ggtitle("ACE diversity")+labs(y = "ACE div
ersity") + stat_compare_means(mapping = NULL, comparisons = my_comparisons, hide.ns = FALSE, label = "p.signif",
label.x = NULL, label.y = NULL, exact =FALSE)
```

```
## [1] FALSE
```

```
compare_means(formula = PD~asthma_rhinitis1,data = diversityRAREF_Q1, method = "wilcox.test", exact= FALSE)
```

```
## # A tibble: 6 × 8
##   .y.   group1 group2             p      p.adj p.format p.signif method
##   <chr> <chr>  <chr>          <dbl>      <dbl> <chr>    <chr>    <chr>
## 1 PD    ARAS   AR     0.486         1          0.49     ns       Wilcoxon
## 2 PD    ARAS   AS     0.418         1          0.42     ns       Wilcoxon
## 3 PD    ARAS   CT     0.0000000303 0.00000018 3.0e-08  ****     Wilcoxon
## 4 PD    AR     AS     0.493         1          0.49     ns       Wilcoxon
## 5 PD    AR     CT     0.00000549   0.000027   5.5e-06  ****     Wilcoxon
## 6 PD    AS     CT     0.272         1          0.27     ns       Wilcoxon
```

```
phyl <- ggplot(diversityRAREF_Q1, aes(factor(asthma_rhinitis1), PD))
phyl2<- phyl + geom_boxplot(aes(fill = factor(asthma_rhinitis1)),outlier.colour = "black", outlier.size = 0.2)+ g
eom_jitter(size=0.2,shape=1)+panel_border(colour = "black", size = 0.5)+ ggtitle("Phylogenetic diversity")+labs(y
= "Phylogenetic  diversity") + stat_compare_means(mapping = NULL, comparisons = my_comparisons, hide.ns = FALSE,
label = "p.signif",  label.x = NULL, label.y = NULL, exact =FALSE)
```

```
## [1] FALSE
```

```
plot_grid(chao2, shan2, ACE2, phyl2, nrows=2, cols=2, align = "v")
```

```
## Warning in plot_grid(chao2, shan2, ACE2, phyl2, nrows = 2, cols = 2, align = "v"): Argument
## 'cols' is deprecated. Use 'ncol' instead.
```

```
## Warning: Removed 10 rows containing non-finite values (`stat_boxplot()`).
```

```
## Warning: Removed 10 rows containing non-finite values (`stat_signif()`).
```

```
## Warning: Removed 10 rows containing missing values (`geom_point()`).
```

```
## Warning in as_grob.default(plot): Cannot convert object of class numeric into a grob.
```

```
## Warning: Graphs cannot be vertically aligned unless the axis parameter is set. Placing
## graphs unaligned.
```

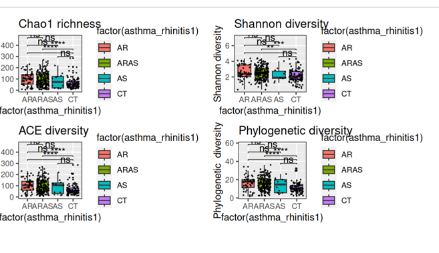

Estimate beta-diversity

Sample pairs

```
ps2 <- subset_samples(ps1, asthma_rhinitis1 == "AR" | asthma_rhinitis1 == "CT"); ps2
```

```
## phyloseq-class experiment-level object
## otu_table()   OTU Table:         [ 6195 taxa and 150 samples ]
## sample_data() Sample Data:       [ 150 samples by 6 sample variables ]
## tax_table()   Taxonomy Table:    [ 6195 taxa by 7 taxonomic ranks ]
## phy_tree()    Phylogenetic Tree: [ 6195 tips and 6194 internal nodes ]
```

```
otuD<-as.data.frame(t(otu_table(ps2)))
diversityRAREF_Q1<-cbind(sample_data(ps2))

uniun<-phyloseq::distance(ps2, method="unifrac")
uniweigh<-phyloseq::distance(ps2, method="wunifrac")
brayd<-phyloseq::distance(ps2, method="bray")
jaccd<-phyloseq::distance(ps2, method="jaccard")

t1<-adonis2(uniun~diversityRAREF_Q1$asthma_rhinitis1, perm=10000); t1
```

```
## Permutation test for adonis under reduced model
## Terms added sequentially (first to last)
## Permutation: free
## Number of permutations: 10000
##
## adonis2(formula = uniun ~ diversityRAREF_Q1$asthma_rhinitis1, permutations = 10000)
##                                     Df SumOfSqs     R2      F    Pr(>F)
## diversityRAREF_Q1$asthma_rhinitis1   1    1.706 0.0491 7.6418 9.999e-05 ***
## Residual                           148   33.039 0.9509
## Total                              149   34.744 1.0000
## ---
## Signif. codes:  0 '***' 0.001 '**' 0.01 '*' 0.05 '.' 0.1 ' ' 1
```

```
t1<-adonis2(uniweigh~diversityRAREF_Q1$asthma_rhinitis1, perm=10000); t1
```

```
## Permutation test for adonis under reduced model
## Terms added sequentially (first to last)
## Permutation: free
## Number of permutations: 10000
##
## adonis2(formula = uniweigh ~ diversityRAREF_Q1$asthma_rhinitis1, permutations = 10000)
##                                     Df SumOfSqs      R2      F    Pr(>F)
## diversityRAREF_Q1$asthma_rhinitis1   1   0.6840 0.09276 15.132 9.999e-05 ***
## Residual                           148   6.6901 0.90724
## Total                              149   7.3741 1.00000
## ---
## Signif. codes:  0 '***' 0.001 '**' 0.01 '*' 0.05 '.' 0.1 ' ' 1
```

```
t1<-adonis2(brayd~diversityRAREF_Q1$asthma_rhinitis1, perm=10000); t1
```

```
## Permutation test for adonis under reduced model
## Terms added sequentially (first to last)
## Permutation: free
## Number of permutations: 10000
##
## adonis2(formula = brayd ~ diversityRAREF_Q1$asthma_rhinitis1, permutations = 10000)
##                                     Df SumOfSqs      R2      F    Pr(>F)
## diversityRAREF_Q1$asthma_rhinitis1   1    2.256 0.04325 6.6905 9.999e-05 ***
## Residual                           148   49.899 0.95675
## Total                              149   52.154 1.00000
## ---
## Signif. codes:  0 '***' 0.001 '**' 0.01 '*' 0.05 '.' 0.1 ' ' 1
```

```
t1<-adonis2(jaccd~diversityRAREF_Q1$asthma_rhinitis1, perm=10000); t1
```

```
## Permutation test for adonis under reduced model
## Terms added sequentially (first to last)
## Permutation: free
## Number of permutations: 10000
##
## adonis2(formula = jaccd ~ diversityRAREF_Q1$asthma_rhinitis1, permutations = 10000)
##                                     Df SumOfSqs      R2      F    Pr(>F)
## diversityRAREF_Q1$asthma_rhinitis1   1    1.849 0.03061 4.6731 9.999e-05 ***
## Residual                           148   58.548 0.96939
## Total                              149   60.397 1.00000
## ---
## Signif. codes:  0 '***' 0.001 '**' 0.01 '*' 0.05 '.' 0.1 ' ' 1
```

All pairs

```
uniun<-phyloseq::distance(ps1, method="unifrac")
uniweigh<-phyloseq::distance(ps1, method="wunifrac")
brayd<-phyloseq::distance(ps1, method="bray")
jaccd<-phyloseq::distance(ps1, method="jaccard")
```

PCoA plots

```
p1 = phyloseq::plot_ordination(ps1, ordinate(ps1, method="PCoA", dist="unifrac", weighted=TRUE), type = "samples"
, color = "asthma_rhinitis1") # label="patient"
p1a=p1 + geom_point(size = 2) + ggtitle("PCoA Weigthed UNIFRAC")
p2 = phyloseq::plot_ordination(ps1, ordinate(ps1, method="PCoA", dist="unifrac"), type = "samples", color = "asth
ma_rhinitis1") # label="patient"
p2a=p2 + geom_point(size = 2) + ggtitle("PCoA Unweigthed UNIFRAC")
p3 = phyloseq::plot_ordination(ps1, ordinate(ps1, method="PCoA", dist="brayd"), type = "samples", color = "asthma
_rhinitis1")
p3a=p3 + geom_point(size = 2) + ggtitle("PCoA Bray-Curtis")
p4 = phyloseq::plot_ordination(ps1, ordinate(ps1, method="PCoA", dist="jaccd"), type = "samples", color = "asthma
_rhinitis1")
p4a=p4 + geom_point(size = 2) + ggtitle("PCoA Jaccard")

plot_grid(p1a, p2a, p3a, p4a, ncol = 2, nrows=2, align = "v")
```

```
## Warning in as_grob.default(plot): Cannot convert object of class numeric into a grob.
```

```
## Warning: Graphs cannot be vertically aligned unless the axis parameter is set. Placing
## graphs unaligned.
```

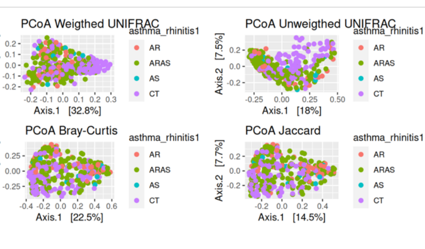

Statistical analyses of taxa % and other variables

```
bygenus <- tax_glom(ps1, taxrank = "Genus") # ASV in taxa_lineage below = genus name
bygenus <- tax_glom(ps1, taxrank = "Phylum") # ASV in taxa_lineage below = phylum name

bygenus.tr <- transform_sample_counts(bygenus, function (x) x/sum(x))
bygenus.tr.f <- filter_taxa(bygenus.tr, function (x) mean(x) > 1e-2, TRUE) # filter taxa below 5%
taxa_names(bygenus.tr.f)
```

```
## [1] "ASV39" "ASV73" "ASV3"  "ASV6"  "ASV2"
```

```
taxa_lineage <- tax_table(bygenus.tr.f);taxa_lineage
```

```
## Taxonomy Table:     [5 taxa by 7 taxonomic ranks]:
##       Kingdom    Phylum             Class Order Family Genus Species
## ASV39 "Bacteria" "Fusobacteriota"   NA    NA    NA     NA    NA
## ASV73 "Bacteria" "Bacteroidota"     NA    NA    NA     NA    NA
## ASV3  "Bacteria" "Actinobacteriota" NA    NA    NA     NA    NA
## ASV6  "Bacteria" "Proteobacteria"   NA    NA    NA     NA    NA
## ASV2  "Bacteria" "Firmicutes"       NA    NA    NA     NA    NA
```

```
taxa_abun<-as.data.frame(t(otu_table(bygenus.tr.f)))
taxa_abun1<-cbind(sample_data(ps1),taxa_abun)
View(taxa_abun1)
```

```
my_comparisons <- list(c("AS", "CT"),c("AR", "CT"),c("ARAS", "CT"),c("AS", "AR"),c("AS", "ARAS"),c("AR", "ARAS"))
# List here the group pairs to compare statistically
```

```
library(ggpubr)
```

Run text for each dominant ASV corresponding to phyla and genera

```
compare_means(formula = ASV39~asthma_rhinitis1, data = taxa_abun1, method = "wilcox.test", exact= FALSE)
```

```
## # A tibble: 6 × 8
##   .y.   group1 group2        p        p.adj p.format p.signif method
##   <chr> <chr>  <chr>     <dbl>        <dbl> <chr>    <chr>    <chr>
## 1 ASV39 ARAS   AR     4.65e- 1 0.55         0.465    ns       Wilcoxon
## 2 ASV39 ARAS   AS     2.75e- 1 0.55         0.275    ns       Wilcoxon
## 3 ASV39 ARAS   CT     4.28e-10 0.0000000026 4.3e-10  ****     Wilcoxon
## 4 ASV39 AR     AS     1.84e- 1 0.55         0.184    ns       Wilcoxon
## 5 ASV39 AR     CT     4.22e- 7 0.0000021    4.2e-07  ****     Wilcoxon
## 6 ASV39 AS     CT     5.15e- 2 0.21         0.051    ns       Wilcoxon
```

PICRUSt2 commands

Bash section

```
place_seqs.py -s ASVs.fasta -o out.tre -p 9 --intermediate intermediate/place_seqs
```

```
hsp.py -i 16S -t ASV_tree.txt -o marker_predicted_and_nsti.tsv.gz -p 9 -n
```

```
hsp.py -i EC -t ASV_tree.txt -o marker_predicted_and_nsti.tsv.gz -p 9 -n
```

```
metagenome_pipeline.py -i ASVs.biom -m marker_predicted_and_nsti.tsv.gz -f EC_predicted.tsv.gz -o EC_metagenome_o
ut --strat_out -p 9
```

```
pathway_pipeline.py -i EC_metagenome_out/pred_metagenome_contrib.tsv.gz -o pathways_out -p 9
```

```
add_descriptions.py -i EC_metagenome_out/pred_metagenome_unstrat.tsv.gz -m EC -o EC_metagenome_out/pred_metagenom
e_unstrat_descrip.tsv.gz
```

```
add_descriptions.py -i pathways_out/path_abun_unstrat.tsv.gz -m METACYC -o pathways_out/path_abun_unstrat_descrip
.tsv.gz
```

End of bash section

```
library("data.table")
library("ComplexHeatmap")
library("RColorBrewer")
library("circlize")
```

Laod pathway table

```
pws_table <- fread("path_abun_unstrat_descrip.tsv.gz")
```

Load phyloseq object

```
nasal_CT_ps <- read_rds("pathway_CT.RDS")
nasal_AR_ps <- read_rds("pathway_AR.RDS")
nasal_ARAS_ps <- read_rds("pathway_ARAS.RDS")
```

```
merged_nasal <- merge_phyloseq(nasal_CT_ps, nasal_AR_ps, nasal_ARAS_ps)

merged_nasal <- subset_samples(merged_nasal, sample_names(merged_nasal) != "NO67")

sample_data(merged_nasal)$sample_code <- gsub("_S.+$","",sample_data(merged_nasal)$sample_code)
```

```
my_design <- ~ sex + age + asthma_rhinitis
```

```
my_pw_counts_nasal <- pws_table %>%
  dplyr::select(pathway, description, sample_data(merged_nasal)$sample_code)


only_cs_my_pw_nasal <- my_pw_counts_nasal[,-c(1:2)]


ds_obj_nasal <- DESeqDataSetFromMatrix(countData = round(only_cs_my_pw_nasal),
                                       colData = sample_data(merged_nasal),
                                       design = my_design)
```

```
## Warning in class(from) <- NULL: Configuración de clase (x) a NULL; resultado ya no será un
## objeto S4
```

```
## converting counts to integer mode
```

```
## Warning in DESeqDataSet(se, design = design, ignoreRank): some variables in design formula
## are characters, converting to factors
```

```
##   the design formula contains one or more numeric variables with integer values,
##   specifying a model with increasing fold change for higher values.
##   did you mean for this to be a factor? if so, first convert
##   this variable to a factor using the factor() function
```

```
##   the design formula contains one or more numeric variables that have mean or
##   standard deviation larger than 5 (an arbitrary threshold to trigger this message).
##   Including numeric variables with large mean can induce collinearity with the intercept.
##   Users should center and scale numeric variables in the design to improve GLM convergence.
```

```
ds_nasal_analysis <- DESeq(ds_obj_nasal)
```

```
## estimating size factors
```

```
## estimating dispersions
```

```
## gene-wise dispersion estimates
```

```
## mean-dispersion relationship
```

```
## -- note: fitType='parametric', but the dispersion trend was not well captured by the
##    function: y = a/x + b, and a local regression fit was automatically substituted.
##    specify fitType='local' or 'mean' to avoid this message next time.
```

```
## final dispersion estimates
```

```
## fitting model and testing
```

```
## -- replacing outliers and refitting for 6 genes
## -- DESeq argument 'minReplicatesForReplace' = 7
## -- original counts are preserved in counts(dds)
```

```
## estimating dispersions
```

```
## fitting model and testing
```

```
ARAS_vs_CT_wald <- results(ds_nasal_analysis, contrast = c("asthma_rhinitis", "AR", "NO"))

AR_vs_CT_wald <- results(ds_nasal_analysis, contrast = c("asthma_rhinitis", "RN", "NO"))

ARAS_vs_AR_wald <- results(ds_nasal_analysis, contrast = c("asthma_rhinitis", "AR", "RN"))
```

Subset for p-value < 0.05

```
nasal_ARAS_CT_sigres <- ARAS_vs_CT_wald %>%
  data.frame() %>%
  rownames_to_column(var = "pathway") %>%
  as_tibble() %>%
  dplyr::filter(padj < 0.05)
```

```
nasal_AR_CT_sigres <- AR_vs_CT_wald %>%
  data.frame() %>%
  rownames_to_column(var = "pathway") %>%
  as_tibble() %>%
  dplyr::filter(padj < 0.05)
```

```
nasal_ARAS_vs_AR_sigres <- ARAS_vs_AR_wald %>%
```

```r
  data.frame() %>%
  rownames_to_column(var = "pathway") %>%
  as_tibble() %>%
  dplyr::filter(padj < 0.05)
```

```r
nasal_ARAS_CT_sigres$pathway <- pws_table$description[as.numeric(nasal_ARAS_CT_sigres$pathway)]


nasal_ARAS_CT_sigres <- nasal_ARAS_CT_sigres[order(nasal_ARAS_CT_sigres$log2FoldChange, decreasing = TRUE),]


nasal_ARAS_CT_sigres <- nasal_ARAS_CT_sigres[abs(nasal_ARAS_CT_sigres$log2FoldChange) >= 2,]


nasal_ARAS_CT_sigres
```

```
## # A tibble: 72 × 7
##    pathway                                  baseM…¹ log2F…² lfcSE  stat    pvalue      padj
##    <chr>                                      <dbl>   <dbl> <dbl> <dbl>     <dbl>     <dbl>
##  1 purine nucleotides degradation II (aero…   194.     9.37 0.422 22.2  5.03e-109 7.42e-107
##  2 adenosine nucleotides degradation II       164.     9.21 0.439 21.0  8.27e- 98 4.88e- 96
##  3 guanosine nucleotides degradation III      212.     9.21 0.360 25.6  4.75e-144 1.40e-141
##  4 purine nucleobases degradation I (anaer…   149.     8.67 0.397 21.9  7.65e-106 7.52e-104
##  5 superpathway of UDP-N-acetylglucosamine…    84.3    8.47 1.81   4.68 2.88e-  6 8.58e-  6
##  6 myo-, chiro- and scillo-inositol degrad…   280.     8.20 0.406 20.2  1.32e- 90 6.50e- 89
##  7 L-glutamate and L-glutamine biosynthesis   158.     7.75 0.369 21.0  4.99e- 98 3.68e- 96
##  8 superpathway of glucose and xylose degr…   153.     7.48 0.398 18.8  8.44e- 79 3.56e- 77
##  9 cob(II)yrinate a,c-diamide biosynthesis…    85.9    6.89 0.384 17.9  7.60e- 72 1.87e- 70
## 10 cob(II)yrinate a,c-diamide biosynthesis…    48.7    6.81 0.459 14.8  8.68e- 50 1.07e- 48
## # … with 62 more rows, and abbreviated variable names ¹baseMean, ²log2FoldChange
```

```r
nasal_AR_CT_sigres$pathway <- pws_table$description[as.numeric(nasal_AR_CT_sigres$pathway)]

nasal_AR_CT_sigres <- nasal_AR_CT_sigres[order(nasal_AR_CT_sigres$log2FoldChange, decreasing = TRUE),]

nasal_AR_CT_sigres <- nasal_AR_CT_sigres[abs(nasal_AR_CT_sigres$log2FoldChange) >= 2,]

nasal_AR_CT_sigres
```

```
## # A tibble: 72 × 7
##    pathway                                  baseM…¹ log2F…² lfcSE  stat    pvalue     padj
##    <chr>                                      <dbl>   <dbl> <dbl> <dbl>     <dbl>    <dbl>
##  1 guanosine nucleotides degradation III      212.     9.13 0.427 21.4  1.56e-101 4.61e-99
##  2 octane oxidation                            76.0    9.03 0.495 18.3  1.92e- 74 2.83e-72
##  3 adenosine nucleotides degradation II       164.     8.99 0.531 16.9  2.06e- 64 6.75e-63
##  4 purine nucleotides degradation II (aerob…  194.     8.92 0.509 17.5  1.01e- 68 4.95e-67
##  5 myo-, chiro- and scillo-inositol degrada…  280.     8.48 0.491 17.3  7.28e- 67 3.07e-65
##  6 L-tyrosine degradation I                    51.6    8.43 0.536 15.7  1.01e- 55 2.72e-54
##  7 purine nucleobases degradation I (anaero…  149.     8.34 0.475 17.5  6.99e- 69 4.12e-67
##  8 superpathway of UDP-N-acetylglucosamine-…   84.3    8.24 2.28   3.61 3.09e-  4 1.13e- 3
##  9 superpathway of glucose and xylose degra…  153.     8.15 0.478 17.0  4.63e- 65 1.71e-63
## 10 cob(II)yrinate a,c-diamide biosynthesis …   48.7    7.98 0.556 14.3  1.26e- 46 2.55e-45
## # … with 62 more rows, and abbreviated variable names ¹baseMean, ²log2FoldChange
```

```r
nasal_ARAS_vs_AR_sigres$pathway <- pws_table$description[as.numeric(nasal_ARAS_vs_AR_sigres$pathway)]
nasal_ARAS_vs_AR_sigres <- nasal_ARAS_vs_AR_sigres[order(nasal_ARAS_vs_AR_sigres$log2FoldChange,
                                                  decreasing = T),]

nasal_ARAS_vs_AR_sigres <- nasal_ARAS_vs_AR_sigres[abs(nasal_ARAS_vs_AR_sigres$log2FoldChange) >= 2,]
nasal_ARAS_vs_AR_sigres
```

```
## # A tibble: 18 × 7
##    pathway                                  baseM…¹ log2F…² lfcSE   stat   pvalue     padj
##    <chr>                                      <dbl>   <dbl> <dbl>  <dbl>    <dbl>    <dbl>
##  1 L-valine degradation I                    55.6      8.54 0.658  13.0  1.57e-38 2.32e-36
##  2 CMP-legionaminate biosynthesis I          11.5      7.84 0.621  12.6  1.69e-36 1.66e-34
##  3 4-hydroxyphenylacetate degradation        19.1     -2.03 0.417  -4.86 1.15e- 6 1.17e- 5
##  4 pyridoxal 5'-phosphate biosynthesis I    144.      -2.06 0.308  -6.69 2.17e-11 5.82e-10
##  5 fatty acid salvage                       205.      -2.12 0.328  -6.47 9.98e-11 2.45e- 9
##  6 aromatic biogenic amine degradation (bac…  16.1    -2.24 0.430  -5.22 1.80e- 7 2.13e- 6
##  7 glucose degradation (oxidative)           14.0     -2.27 0.428  -5.30 1.13e- 7 1.43e- 6
##  8 nicotinate degradation I                  13.6     -2.28 0.429  -5.32 1.06e- 7 1.43e- 6
##  9 norspermidine biosynthesis                68.1     -2.91 0.302  -9.62 6.43e-22 2.37e-20
## 10 octane oxidation                          76.0     -3.85 0.339 -11.4  6.36e-30 3.75e-28
## 11 L-tyrosine degradation I                  51.6     -4.40 0.374 -11.8  6.48e-32 4.78e-30
## 12 superpathway of glycerol degradation to …  0.495   -4.93 0.990  -4.98 6.45e- 7 7.32e- 6
## 13 succinate fermentation to butanoate        1.80    -6.70 1.21   -5.51 3.55e- 8 6.55e- 7
## 14 L-lysine fermentation to acetate and but…  2.02    -6.92 0.906  -7.64 2.19e-14 6.46e-13
## 15 acetyl-CoA fermentation to butanoate II    6.66    -7.50 0.703 -10.7  1.57e-26 7.70e-25
## 16 pyruvate fermentation to acetone           4.96    -8.19 0.897  -9.13 6.92e-20 2.27e-18
## 17 L-glutamate degradation V (via hydroxygl… 13.4     -8.59 0.612 -14.0  1.01e-44 2.98e-42
## 18 glycerol degradation to butanol           11.9     -9.42 0.907 -10.4  2.95e-25 1.24e-23
## # … with abbreviated variable names ¹baseMean, ²log2FoldChange
```

From these last objects, prepare log2foldchange vectors

Log2fc for ARAS vs CT

```r
log2fc_nasal_ARAS_CT <- nasal_ARAS_CT_sigres$log2FoldChange %>%
  as.matrix()


colnames(log2fc_nasal_ARAS_CT) <- "log2FC"
```

```r
log2fc_nasal_ARAS_CT_colors <- colorRamp2(c(min(log2fc_nasal_ARAS_CT),0,max(log2fc_nasal_ARAS_CT)),
                                     c("blue","white","orange"))



hm_nasal_CT_ARAS_fc <- Heatmap(log2fc_nasal_ARAS_CT, cluster_rows = F, row_labels = nasal_ARAS_CT_sigres$pathway,
                        col = log2fc_nasal_ARAS_CT_colors, width = unit(30,"mm"),
                        cell_fun = function(j,i,x,y,w,h,col){
                          grid.text(round(log2fc_nasal_ARAS_CT[i,j],2),x,y)
                        }, name = "log2FC", column_labels = "")



draw(hm_nasal_CT_ARAS_fc, heatmap_legend_side = "left")
```

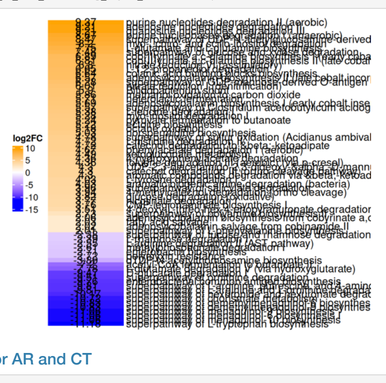

Log2foc for AR and CT

```r
log2fc_nasal_AR_CT <- nasal_AR_CT_sigres$log2FoldChange %>%
  as.matrix()

colnames(log2fc_nasal_AR_CT) <- "log2FC"

log2fc_nasal_AR_CT_colors <- colorRamp2(c(min(log2fc_nasal_AR_CT),0,max(log2fc_nasal_AR_CT)),
                                     c("blue","white","orange"))


hm_nasal_CT_AR_fc <- Heatmap(log2fc_nasal_AR_CT, cluster_rows = F, row_labels = nasal_AR_CT_sigres$pathway,
                        width = unit(30,"mm"), col = log2fc_nasal_AR_CT_colors,
                        cell_fun = function(i,j,x,y,w,h,col){
                          grid.text(round(log2fc_nasal_AR_CT[j,i],2),x,y)
                        }, name = "log2FC", column_labels = "")


draw(hm_nasal_CT_AR_fc, heatmap_legend_side = "left")
```

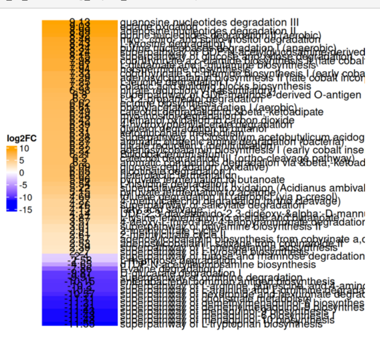

Log2fc for ARAS vs AR

```r
log2fc_nasal_ARAS_AR <- nasal_ARAS_vs_AR_sigres$log2FoldChange %>%
  as.matrix()

colnames(log2fc_nasal_ARAS_AR) <- "log2FC"


log2fc_nasal_ARAS_AR_colors <- colorRamp2(c(min(log2fc_nasal_ARAS_AR),0,max(log2fc_nasal_ARAS_AR)),
                                     c("blue","white","orange"))


hm_nasal_ARAS_AR_fc <- Heatmap(log2fc_nasal_ARAS_AR, cluster_rows = F, row_labels = nasal_ARAS_vs_AR_sigres$pathw
ay,
                        width = unit(30,"mm"), col = log2fc_nasal_ARAS_AR_colors,
                        cell_fun = function(i,j,x,y,w,h,col){
                          grid.text(round(log2fc_nasal_ARAS_AR[j,i],2),x,y)
                        }, name = "log2FC", column_labels = "")


draw(hm_nasal_ARAS_AR_fc, heatmap_legend_side = "left")
```

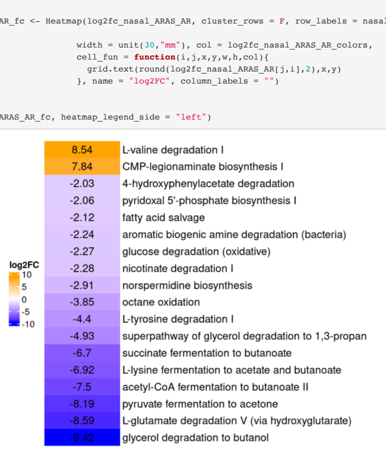

Arrange phyloseq objects for network analysis

```r
nasal_ARAS <- read_rds("pathway_ARAS.RDS")
nasal_AR <- read_rds("pathway_AR.RDS")
nasal_CT <- read_rds("pathway_CT.RDS")
```

```r
library("microbiomeutilities")
```

Define best hit of classification

```r
nasal_ARAS <- format_to_besthit(nasal_ARAS, prefix = "")
nasal_AR <- format_to_besthit(nasal_AR, prefix = "")
nasal_CT <- format_to_besthit(nasal_CT, prefix = "")
```

Agglomerate taxa at species level

```r
nasal_ARAS <- tax_glom(nasal_ARAS, taxrank = rank_names(nasal_ARAS)[7])
nasal_AR <- tax_glom(nasal_AR, taxrank = rank_names(nasal_AR)[7])
nasal_CT <- tax_glom(nasal_CT, taxrank = rank_names(nasal_CT)[7])
```

Filter out low prevalent taxa

```r
library("metagMisc")
```

```r
nasal_ARASprv <- phyloseq_filter_prevalence(nasal_ARAS, prev.trh = 0.10 , abund.trh = 3,
                                            threshold_condition = "OR", abund.type = "median")

nasal_ARprv <- phyloseq_filter_prevalence(nasal_AR, prev.trh = 0.10 , abund.trh = 3,
                                            threshold_condition = "OR", abund.type = "median")

nasal_CTprv <- phyloseq_filter_prevalence(nasal_CT, prev.trh = 0.10 , abund.trh = 3,
                                            threshold_condition = "OR", abund.type = "median" )
```

Remove non prokaryotic ASVs

```r
taxa_forfilter <- c("Chloroplast", "Mitochondria","Eukaryota")

ps_list = list(nasal_ARASprv, nasal_ARprv, nasal_CTprv)

for (i in 1:length(ps_list)) {
  ps_list[[i]] <- subset_taxa(ps_list[[i]],
                              !Domain %in% taxa_forfilter &
                               !Phylum %in% taxa_forfilter &
                               !Class %in% taxa_forfilter &
                               !Order %in% taxa_forfilter &
                               !Family %in% taxa_forfilter &
                               !Genus %in% taxa_forfilter )   }

nasalARAS <- ps_list[[1]]
nasalAR <- ps_list[[2]]
nasalCT <- ps_list[[3]]
```

```r
library("SpiecEasi")
```

Networks calculation using neighborhood selection (mb) method

```r
nasalCT_net = spiec.easi(nasalCT, method = "mb", nlambda = 20,
                         lambda.min.ratio = 1e-2, pulsar.params = list(rep.num=50,
                                                                       ncores=11))
```

```
## Applying data transformations...
```

```
## Selecting model with pulsar using stars...
```

```
## Fitting final estimate with mb...
```

```
## done
```

```r
nasalARAS_net = spiec.easi(nasalARAS, method = "mb", nlambda = 20,
                         lambda.min.ratio = 1e-2, pulsar.params = list(rep.num=50,
                                                                       ncores=11))
```

```
## Applying data transformations...
```

```
## Selecting model with pulsar using stars...
```

```
## Fitting final estimate with mb...
```

```
## done
```

```r
nasalAR_net = spiec.easi(nasalAR, method = "mb", nlambda = 20,
                         lambda.min.ratio = 1e-2, pulsar.params = list(rep.num=50,
                                                                       ncores=11))
```

```
## Applying data transformations...
```

```
## Selecting model with pulsar using stars...
```

```
## Fitting final estimate with mb...
```

```
## done
```

```r
# the resulting variables were stored in RDS format and exported for local analysis
```

Retrieve adjacency matrices

```r
library("NetCoMi")
```

```r
adj_nasalARAS <- symBeta(getOptBeta(nasalARAS_net), mode = "maxabs") %>%
  as.matrix()

rownames(adj_nasalARAS) <- colnames(otu_table(nasalARAS))
colnames(adj_nasalARAS) <- colnames(otu_table(nasalARAS))
```

```r
adj_nasalAR <- symBeta(getOptBeta(nasalAR_net), mode = "maxabs") %>%
  as.matrix()

rownames(adj_nasalAR) <- colnames(otu_table(nasalAR))
colnames(adj_nasalAR) <- colnames(otu_table(nasalAR))
```

```r
adj_nasalCT <- symBeta(getOptBeta(nasalCT_net), mode = "maxabs") %>%
  as.matrix()

rownames(adj_nasalCT) <- colnames(otu_table(nasalCT))
colnames(adj_nasalCT) <- colnames(otu_table(nasalCT))
```

Construct the networks

```r
nasalARAS_netcomi <- netConstruct(data = adj_nasalARAS,
                                  normMethod = "none", zeroMethod = "none",
                                  sparsMethod = "none", dataType = "condDependence",
                                  verbose = 3, seed = 1234)
```

```
## Checking input arguments ... Done.
```

```r
nasalAR_netcomi <- netConstruct(data = adj_nasalAR,
                                  normMethod = "none", zeroMethod = "none",
                                  sparsMethod = "none", dataType = "condDependence",
                                  verbose = 3, seed = 1234)
```

```
## Checking input arguments ... Done.
```

```r
nasalCT_netcomi <- netConstruct(data = adj_nasalCT,
                                  normMethod = "none", zeroMethod = "none",
                                  sparsMethod = "none", dataType = "condDependence",
                                  verbose = 3, seed = 1234)
```

```
## Checking input arguments ... Done.
```

Analyze the networks

```r
nasalARAS_analyzed <- netAnalyze(nasalARAS_netcomi, centrLCC = FALSE, avDissIgnoreInf = TRUE,
                                 hubPar = "degree", hubQuant = 0.90,
                                 normDeg = TRUE, normBetw = TRUE, normEigen = TRUE, verbose = 2)
```

```
## Checking input arguments ... Done.
## Create igraph objects ... Done.
## Compute clustering (cluster_fast_greedy) ... Done.
## Compute shortest paths ... Done.
##
## Compute global properties:
## Average dissimilarity ... Done.
## Edge / vertex connectivity ... Done.
## Natural connectivity ... Done.
## Clustering coefficient ... Done.
## Modularity ... Done.
## Density ... Done.
## P-N-Ratio ... Done.
##
## Compute centralities:
## Degree ... Done.
## Betweenness centrality ... Done.
## Closeness centrality ... Done.
## Eigenvector centrality ... Done.
##
## Compute hubs (based on empirical quantiles) ... Done.
## Compute GCM ... Done.
```

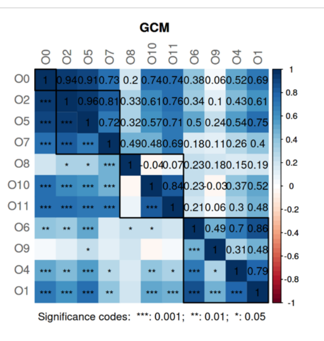


```r
nasalAR_analyzed <- netAnalyze(nasalAR_netcomi, centrLCC = FALSE, avDissIgnoreInf = TRUE,
                                 hubPar = "degree", hubQuant = 0.90,
                                 normDeg = TRUE, normBetw = TRUE, normEigen = TRUE, verbose = 2)
```

```
## Checking input arguments ... Done.
## Create igraph objects ... Done.
## Compute clustering (cluster_fast_greedy) ... Done.
## Compute shortest paths ... Done.
##
## Compute global properties:
## Average dissimilarity ... Done.
## Edge / vertex connectivity ... Done.
## Natural connectivity ... Done.
## Clustering coefficient ... Done.
## Modularity ... Done.
## Density ... Done.
## P-N-Ratio ... Done.
##
## Compute centralities:
## Degree ... Done.
## Betweenness centrality ... Done.
## Closeness centrality ... Done.
## Eigenvector centrality ... Done.
##
## Compute hubs (based on empirical quantiles) ... Done.
## Compute GCM ... Done.
```

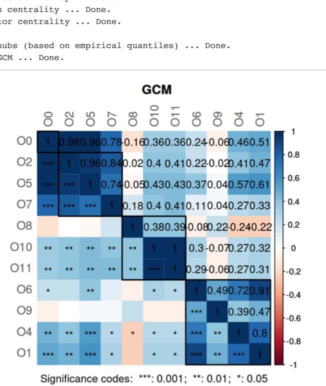


```r
nasalCT_analyzed <- netAnalyze(nasalCT_netcomi, centrLCC = FALSE, avDissIgnoreInf = TRUE,
                                 hubPar = "degree", hubQuant = 0.90,
                                 normDeg = TRUE, normBetw = TRUE, normEigen = TRUE, verbose = 2)
```

```
## Checking input arguments ... Done.
## Create igraph objects ... Done.
## Compute clustering (cluster_fast_greedy) ... Done.
## Compute shortest paths ... Done.
##
## Compute global properties:
## Average dissimilarity ... Done.
## Edge / vertex connectivity ... Done.
## Natural connectivity ... Done.
## Clustering coefficient ... Done.
## Modularity ... Done.
## Density ... Done.
## P-N-Ratio ... Done.
##
## Compute centralities:
## Degree ... Done.
## Betweenness centrality ... Done.
## Closeness centrality ... Done.
## Eigenvector centrality ... Done.
##
## Compute hubs (based on empirical quantiles) ... Done.
## Compute GCM ... Done.
```

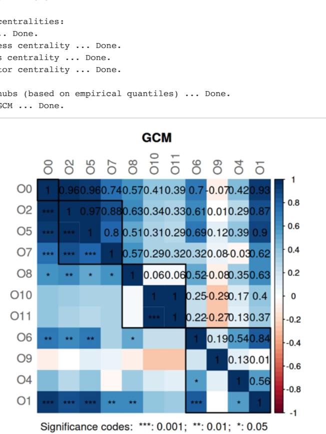

Label the networks nodes by the best hit and remove unwanted characters

Nasal ARAS labels

```r
tax_nasalARAS <- tax_table(nasalARAS) %>%
  as.data.frame() %>%
  dplyr::mutate(lab = paste(Genus, Species, sep = " ")) %>%
  dplyr::select(-best_hit)

tax_nasalARAS$lab <- gsub("g__.+$", "", tax_nasalARAS$lab)
tax_nasalARAS$lab <- gsub("f__.*?\\s", "", tax_nasalARAS$lab)
tax_nasalARAS$lab <- gsub("f__","", tax_nasalARAS$lab)
tax_nasalARAS$lab <- gsub("o__.*?\\s", "", tax_nasalARAS$lab)
tax_nasalARAS$lab <- gsub("o__", "", tax_nasalARAS$lab)



labels_nasalARAS <- tax_nasalARAS$lab
names(labels_nasalARAS) <- rownames(tax_nasalARAS)
```

Nasal AR labels

```r
tax_nasalAR <- tax_table(nasalAR) %>%
  as.data.frame() %>%
  dplyr::mutate(lab = paste(Genus, Species, sep = " ")) %>%
  dplyr::select(-best_hit)

tax_nasalAR$lab <- gsub("g__.+$", "", tax_nasalAR$lab)
tax_nasalAR$lab <- gsub("f__.*?\\s", "", tax_nasalAR$lab)
tax_nasalAR$lab <- gsub("f__","", tax_nasalAR$lab)
tax_nasalAR$lab <- gsub("o__.*?\\s", "", tax_nasalAR$lab)
tax_nasalAR$lab <- gsub("o__", "", tax_nasalAR$lab)


labels_nasalAR <- tax_nasalAR$lab
names(labels_nasalAR) <- rownames(tax_nasalAR)
```

Nasal CT labels

```r
tax_nasalCT <- tax_table(nasalCT) %>%
  as.data.frame() %>%
  dplyr::mutate(lab = paste(Genus, Species, sep = " ")) %>%
  dplyr::select(-best_hit)

tax_nasalCT$lab <- gsub("g__.+$", "", tax_nasalCT$lab)
tax_nasalCT$lab <- gsub("f__.*?\\s", "", tax_nasalCT$lab)
tax_nasalCT$lab <- gsub("f__","", tax_nasalCT$lab)
tax_nasalCT$lab <- gsub("o__.*?\\s", "", tax_nasalCT$lab)
tax_nasalCT$lab <- gsub("o__", "", tax_nasalCT$lab)


labels_nasalCT <- tax_nasalCT$lab
names(labels_nasalCT) <- rownames(tax_nasalCT)
```

Plot nasal bacteriome networks and color nodes by clusters/modules

```r
nasalARAS_circle <- plot(nasalARAS_analyzed, cexNodes = 1.3,
                         cexHubLabels = 1.9 , cexLabels = 2.7, cexTitle = 2.5, nodeColor = "cluster",
                         cexHubs = 1.6, rmSingles = "all",
                         repulsion = 2.8,
                         layout = "layout_nicely", labels = labels_nasalARAS)
```

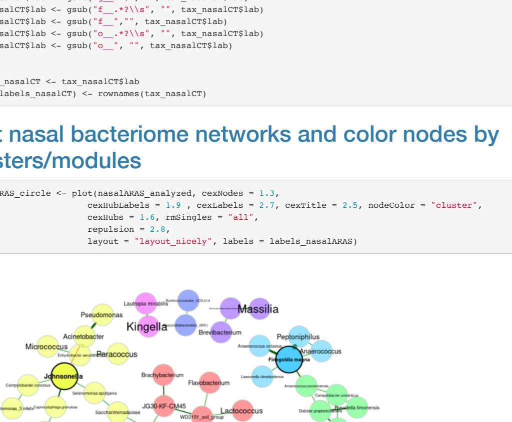

```r
nasalAR_circle <- plot(nasalAR_analyzed, cexNodes = 1.3,
                         cexHubLabels = 1.9 , cexLabels = 2.7, cexTitle = 2.5, nodeColor = "cluster",
                         cexHubs = 1.6, rmSingles = "all",
                         repulsion = 2.8,
                         layout = "layout_nicely", labels = labels_nasalAR)
```

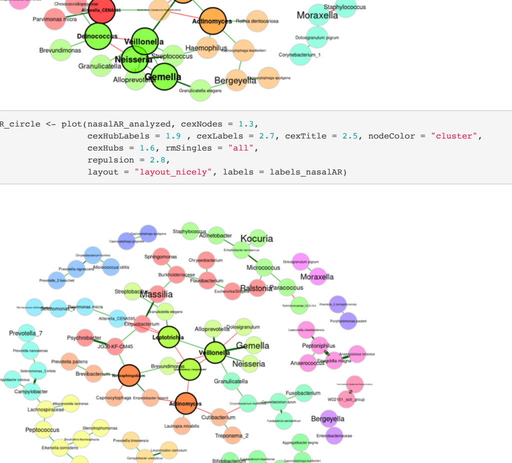

```r
nasalCT_circle <- plot(nasalCT_analyzed, cexNodes = 1.3,
                         cexHubLabels = 1.9 , cexLabels = 2.7, cexTitle = 2.5, nodeColor = "cluster",
                         cexHubs = 1.6, rmSingles = "all",
                         repulsion = 2.8,
                         layout = "layout_nicely", labels = labels_nasalCT )
```

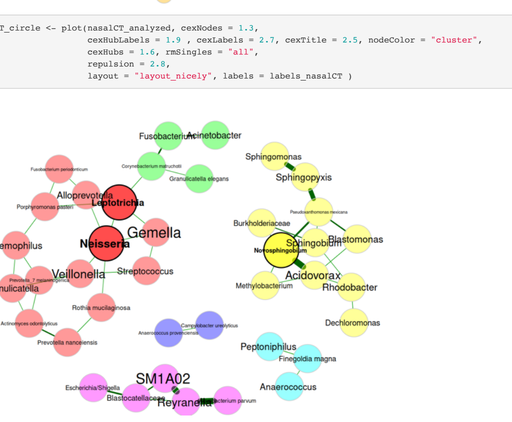

Supplement: Supplementary file 1 [file Figure S1.pdf › Figure S1.pdf]
